# Supplementary material for: Oatk: a de novo assembly tool for complex plant organelle genomes
Source: Genome Biol. 2025 Aug 7;26:235. doi: 10.1186/s13059-025-03676-6 (PMC12329965; doi:10.1186/s13059-025-03676-6)
Supplement: Supplementary file 2 — Supplementary Material 2. [file 13059_2025_3676_MOESM2_ESM.pdf]

Oatk: a de novo assembly tool for complex plant organelle genomes

Chenxi Zhou[1,2], Max Brown[2,3], Mark Blaxter[2], Darwin Tree of Life Project Consortium[2], Shane A. McCarthy[1,2], and Richard Durbin[1,2,*]

[1]Department of Genetics, University of Cambridge, Downing Street, Cambridge, CB2 3EH, UK
[2]Wellcome Sanger Institute, Wellcome Genome Campus, Hinxton, Cambridge, CB10 1SA, UK
[3]Faculty of Science and Engineering, Anglia Ruskin University, East Road, Cambridge, CB1 1PT, UK
[*]Correspondence: rd109@cam.ac.uk

Supplementary Figures

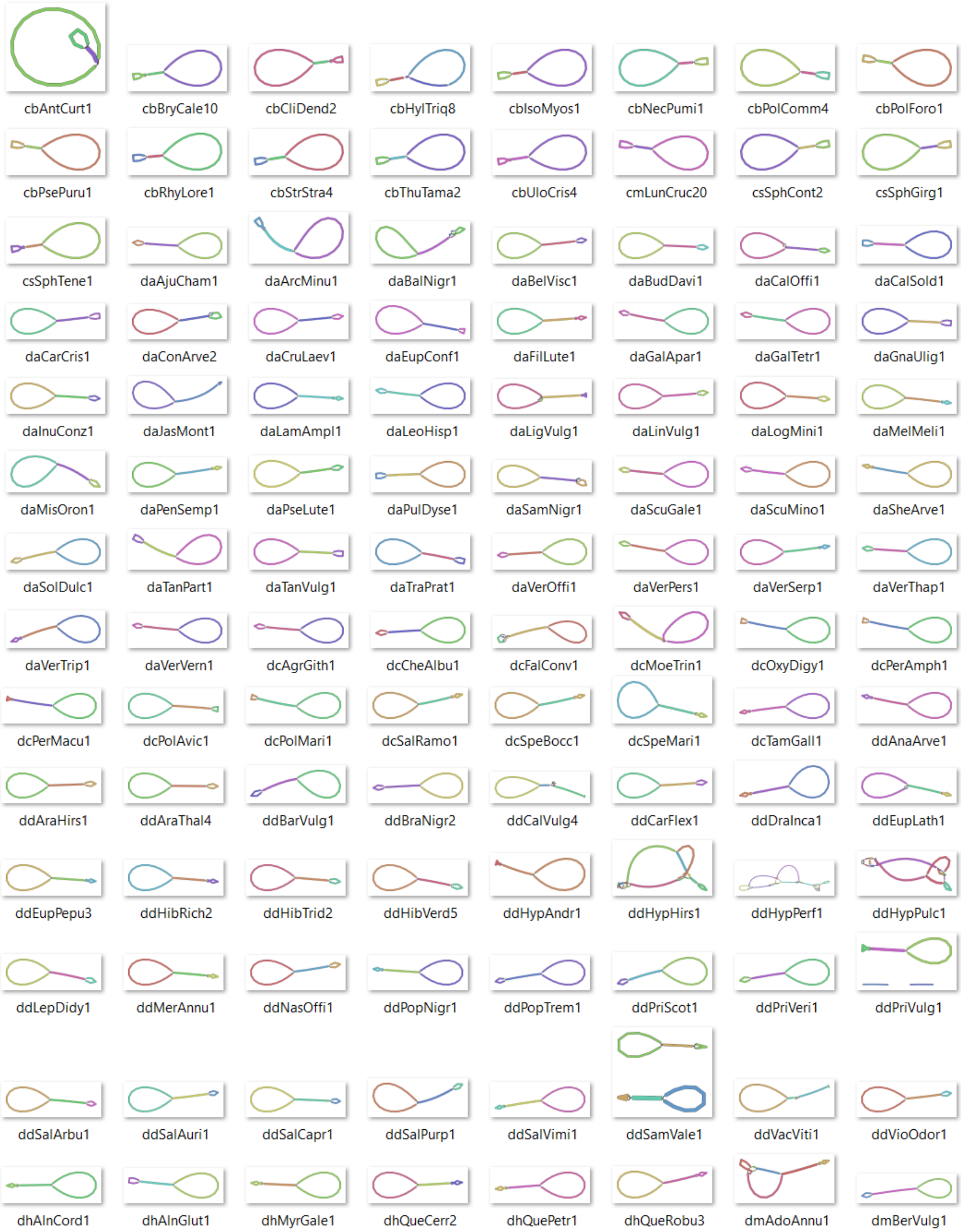

Fig S1. continued on next page

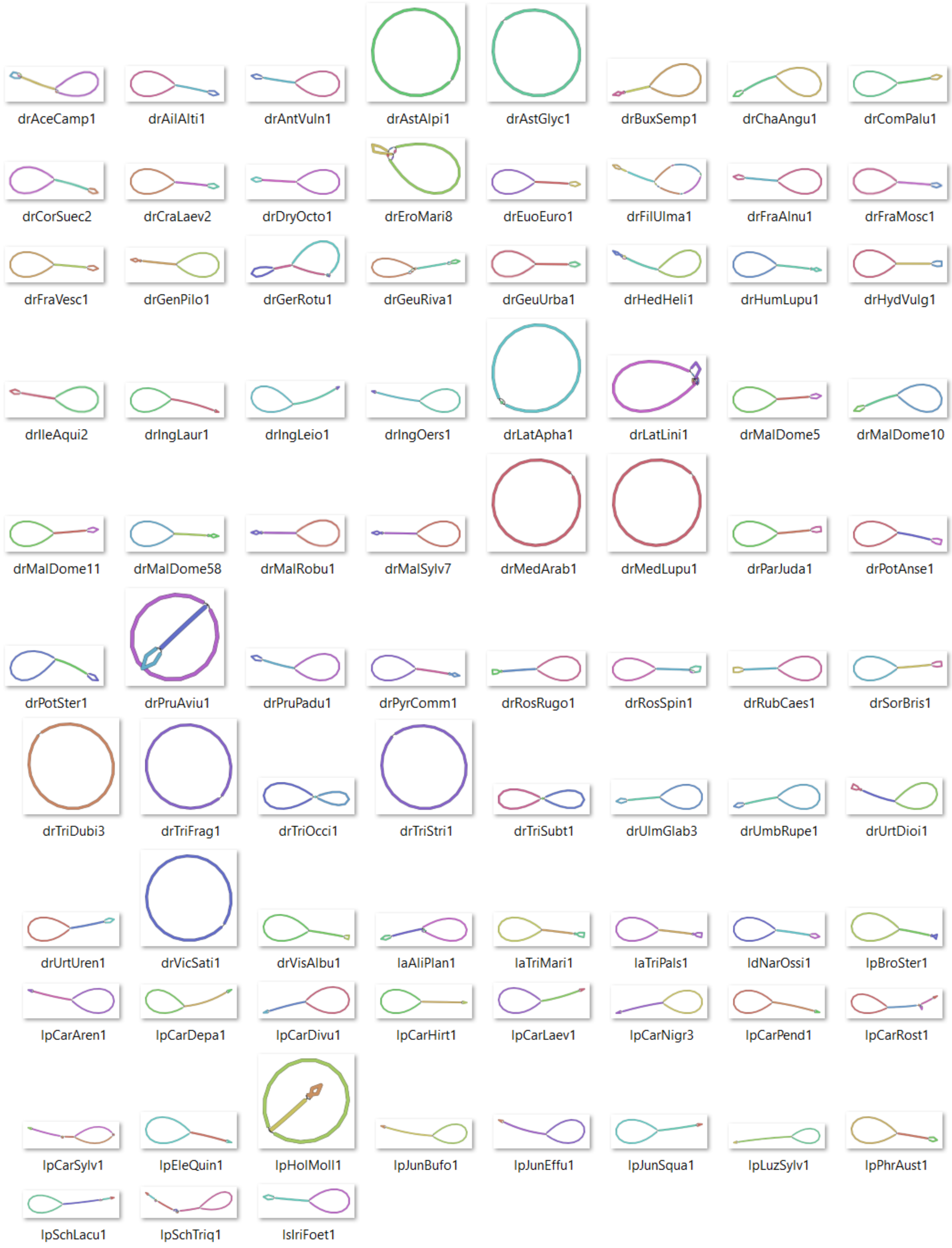

Fig S1. Assembly graphs for all plastomes. The graphs were produced using Bandage.

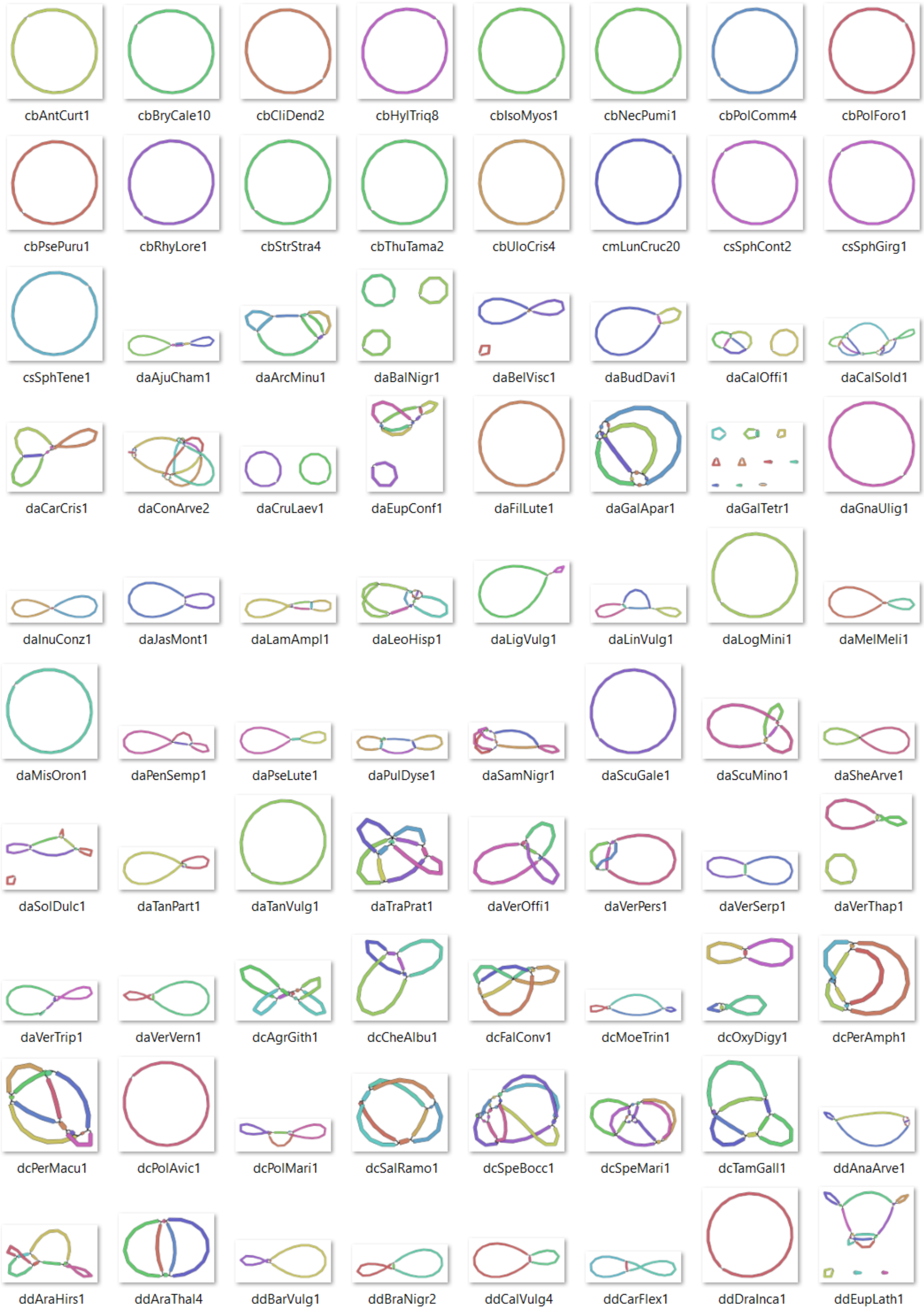

Fig S2. continued on next page

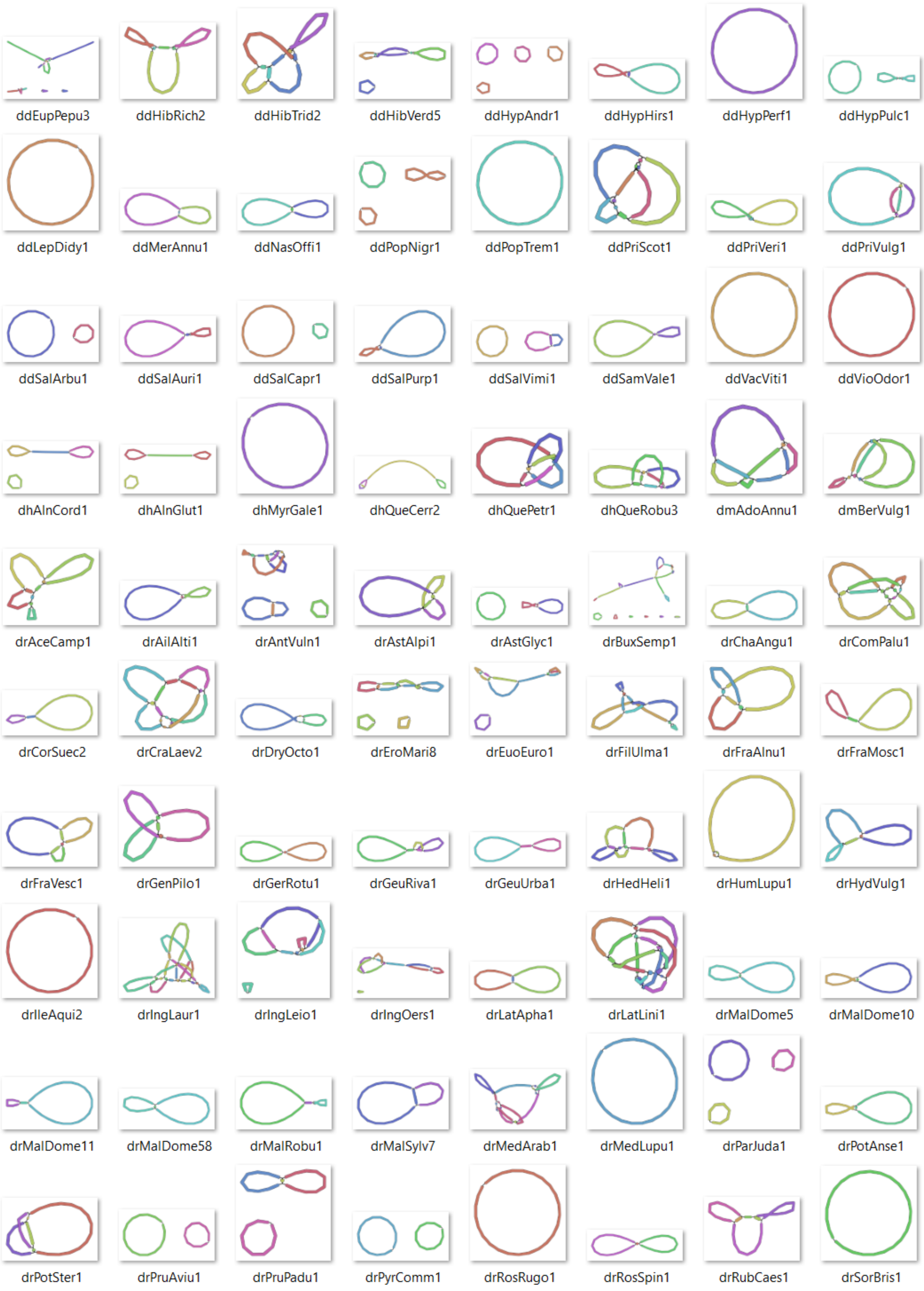

Fig S2. continued on next page

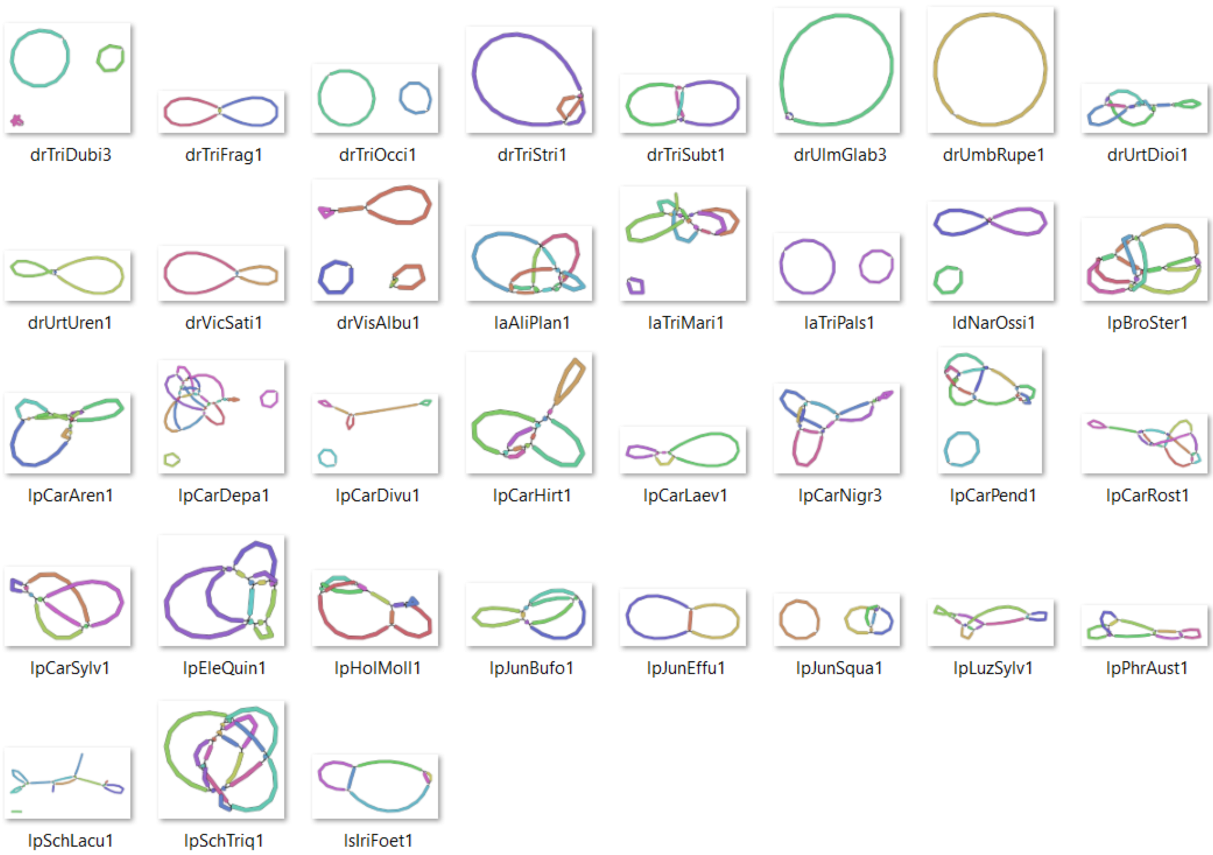

Fig S2. Assembly graphs for all mitogenomes. The graphs were produced using Bandage.

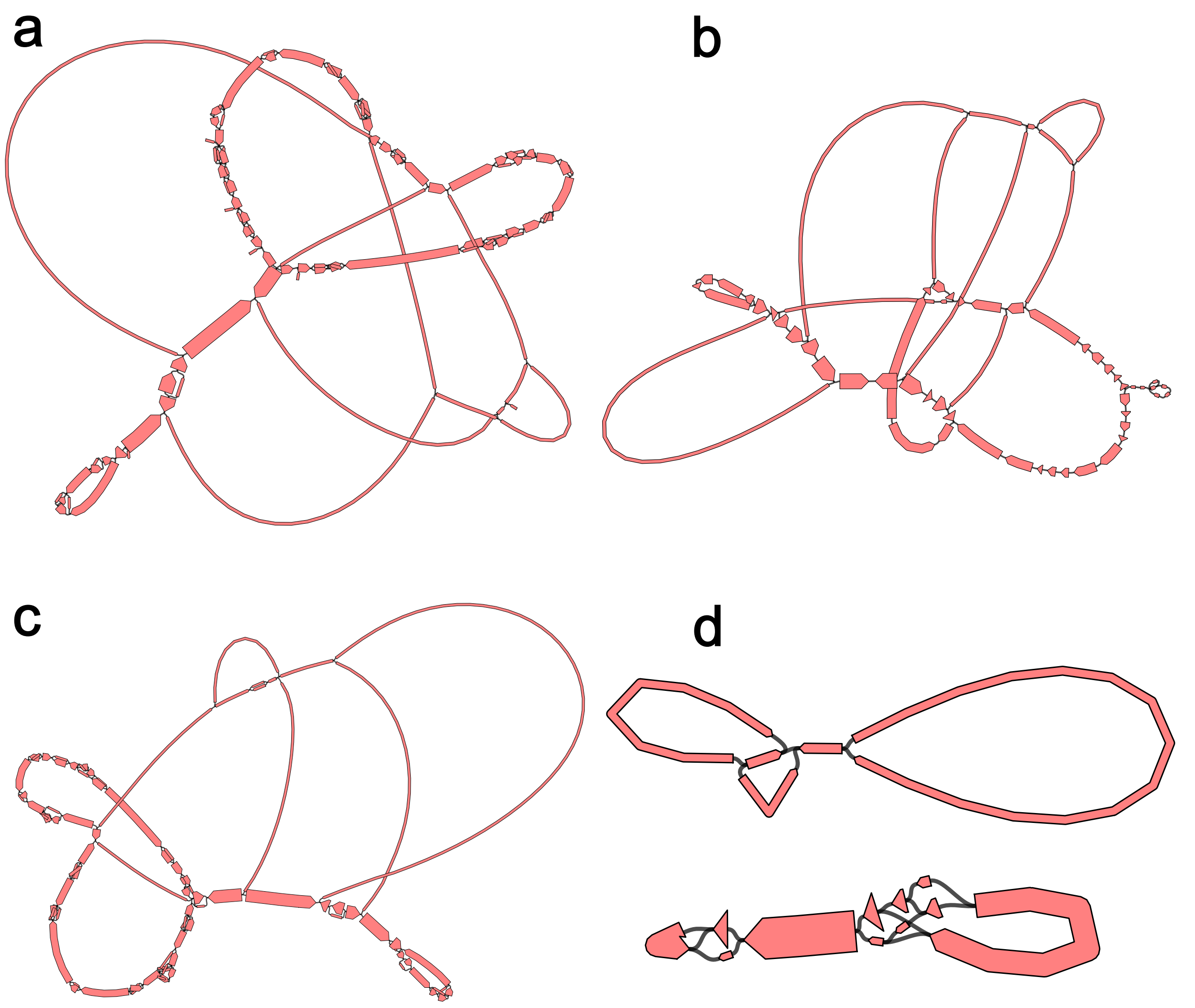

Fig S3. Example mixture assembly graphs generated by different assemblers for *Geum rivale*. a, MBG graph. **b** PMAT graph. **c**, Syncasm raw graph. **d**, Syncasm disentangled graph. In each graph, the width of the bar is proportional to the sequence coverage. The coverage of plastome sequences is higher than that of the mitogenome sequences. The assembly graphs in **a-c** represent mixtures of plastid and mitochondrial genomes. Syncasm successfully separates the two organelle genomes after disentangling (**d**). The assembly graphs were produced using Bandage with additional manual adjustments.

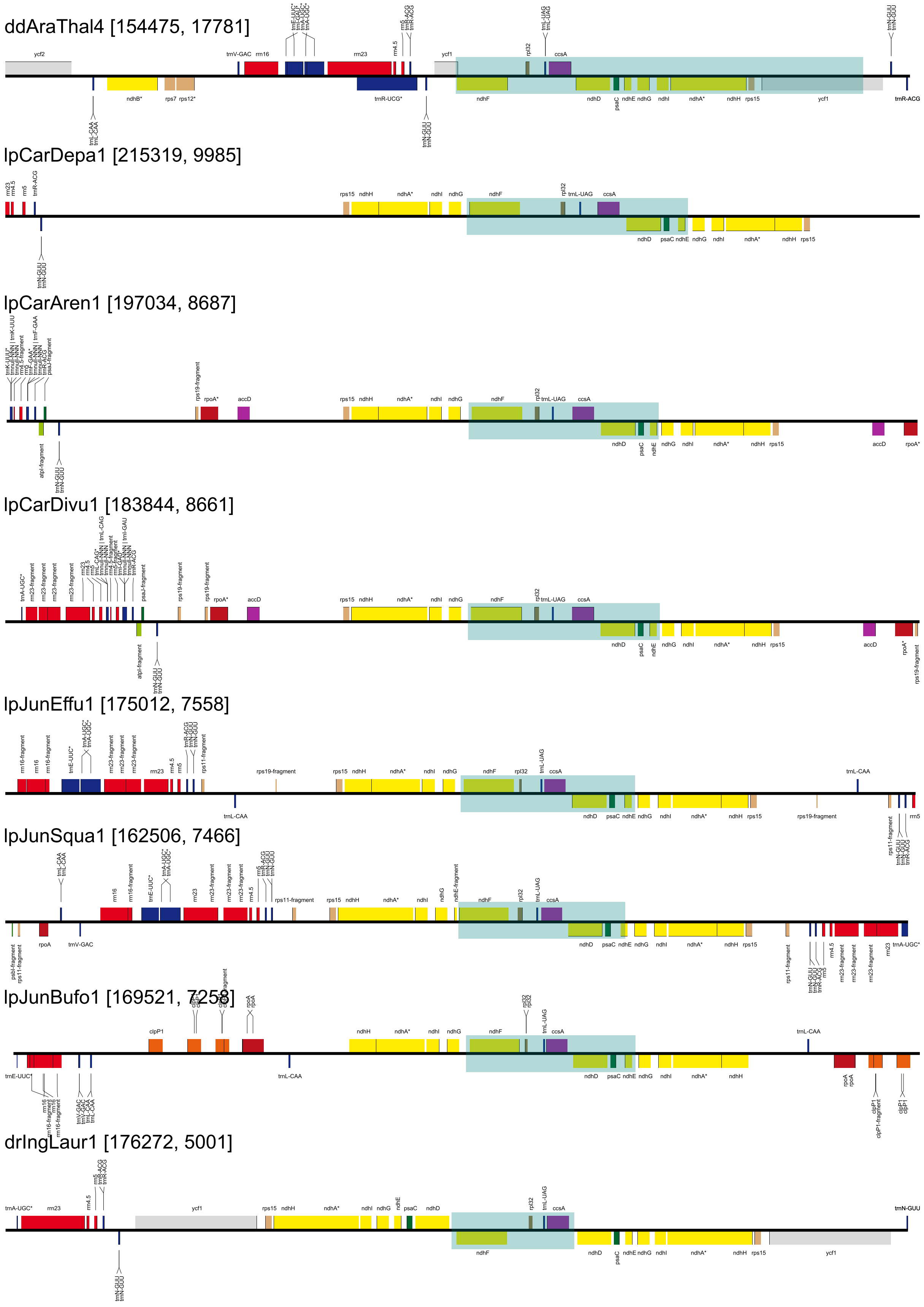

Fig S4. continued on next page

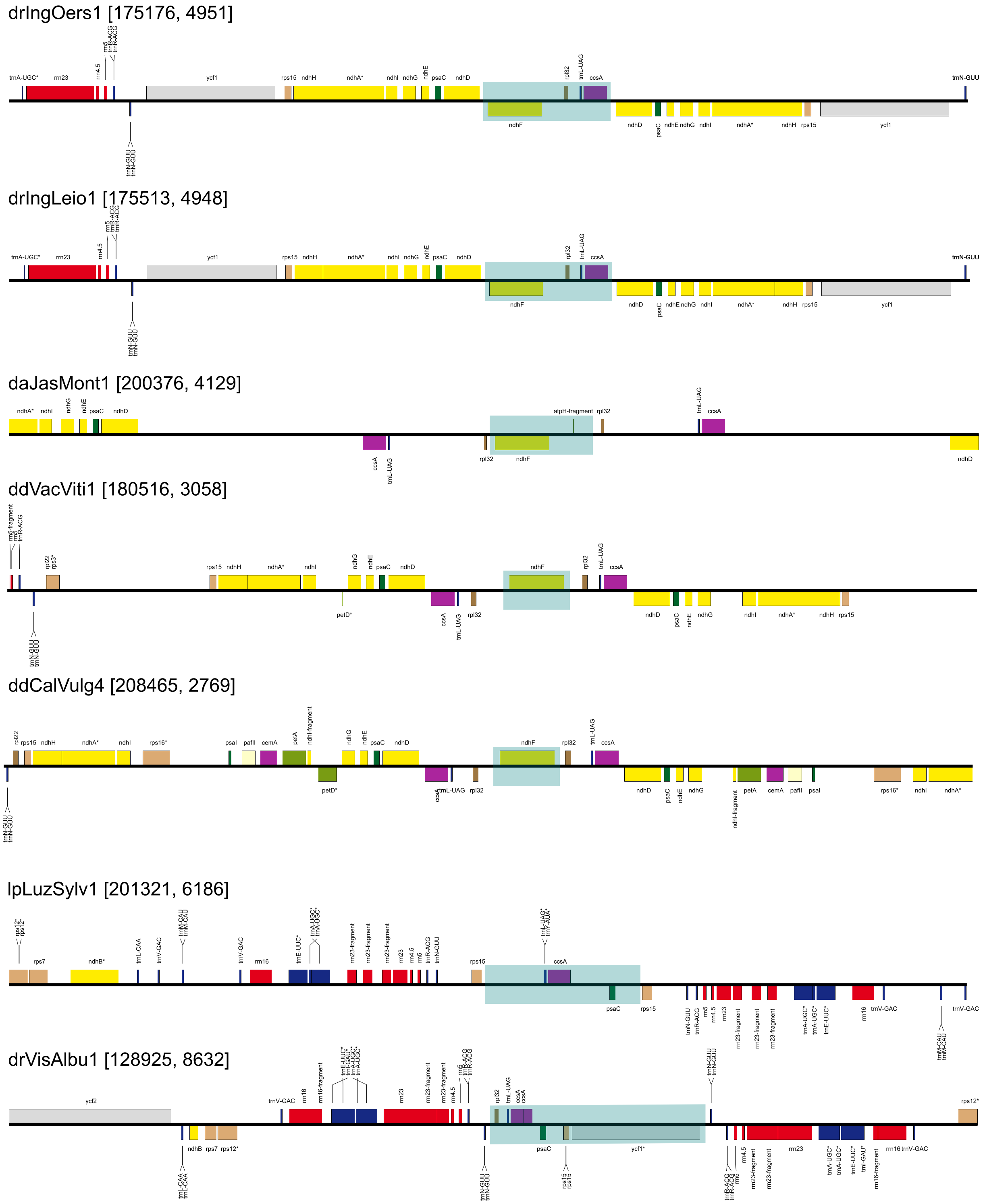

Fig S4. Gene annotation results in the SSC region for species with SSCs smaller than 10 kb. The numbers in square brackets next to the species ID denote the genome size and the SSC size. The light blue box represents the approximate location of the SSC. The annotation was performed using GeSeq and visualised with OGDRAW.

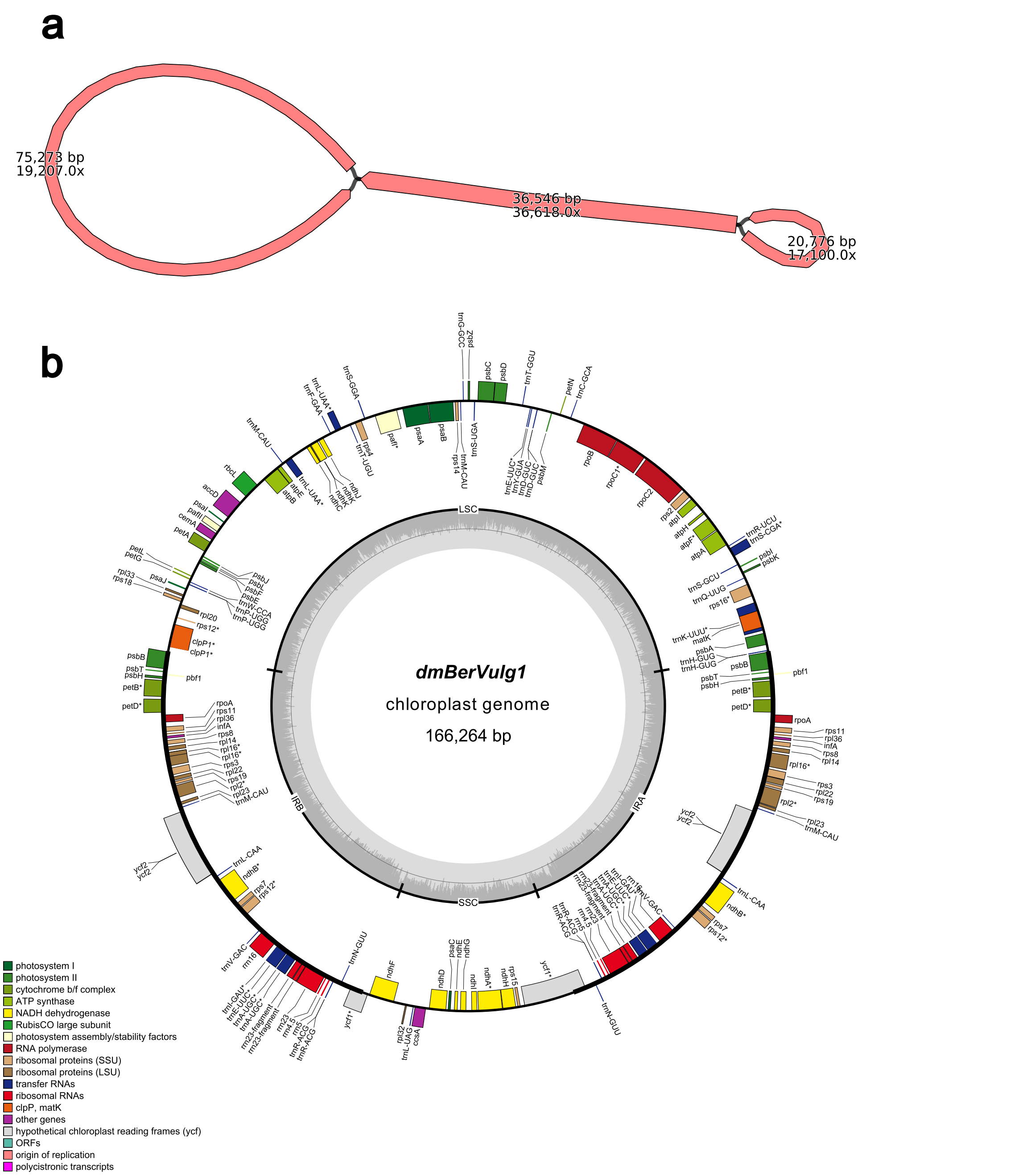

Fig S5. Plastome structure of *Berberis vulgaris*. a, the genome assembly graph. The numbers on the bar indicate the sequence length and coverage. **b**, the genome annotation. The assembly graph was produced using Bandage with additional manual adjustments. The annotation was performed using GeSeq and visualised with OGDRAW.

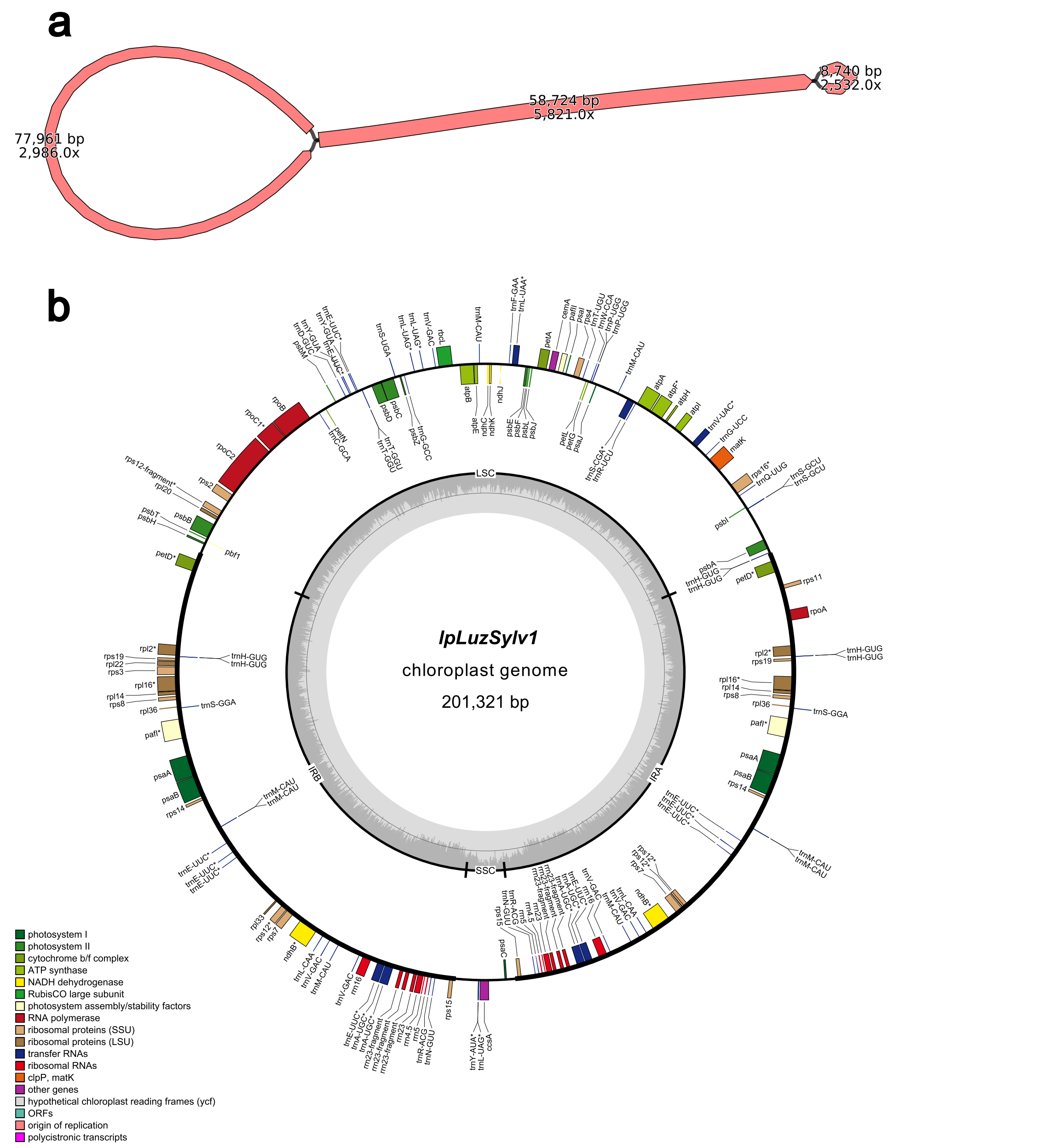

Fig S6. Plastome structure of *Luzula sylvatica*. a, the genome assembly graph. The numbers on the bar indicate the sequence length and coverage. **b**, the genome annotation. The assembly graph was produced using Bandage with additional manual adjustments. The annotation was performed using GeSeq and visualised with OGDRAW.

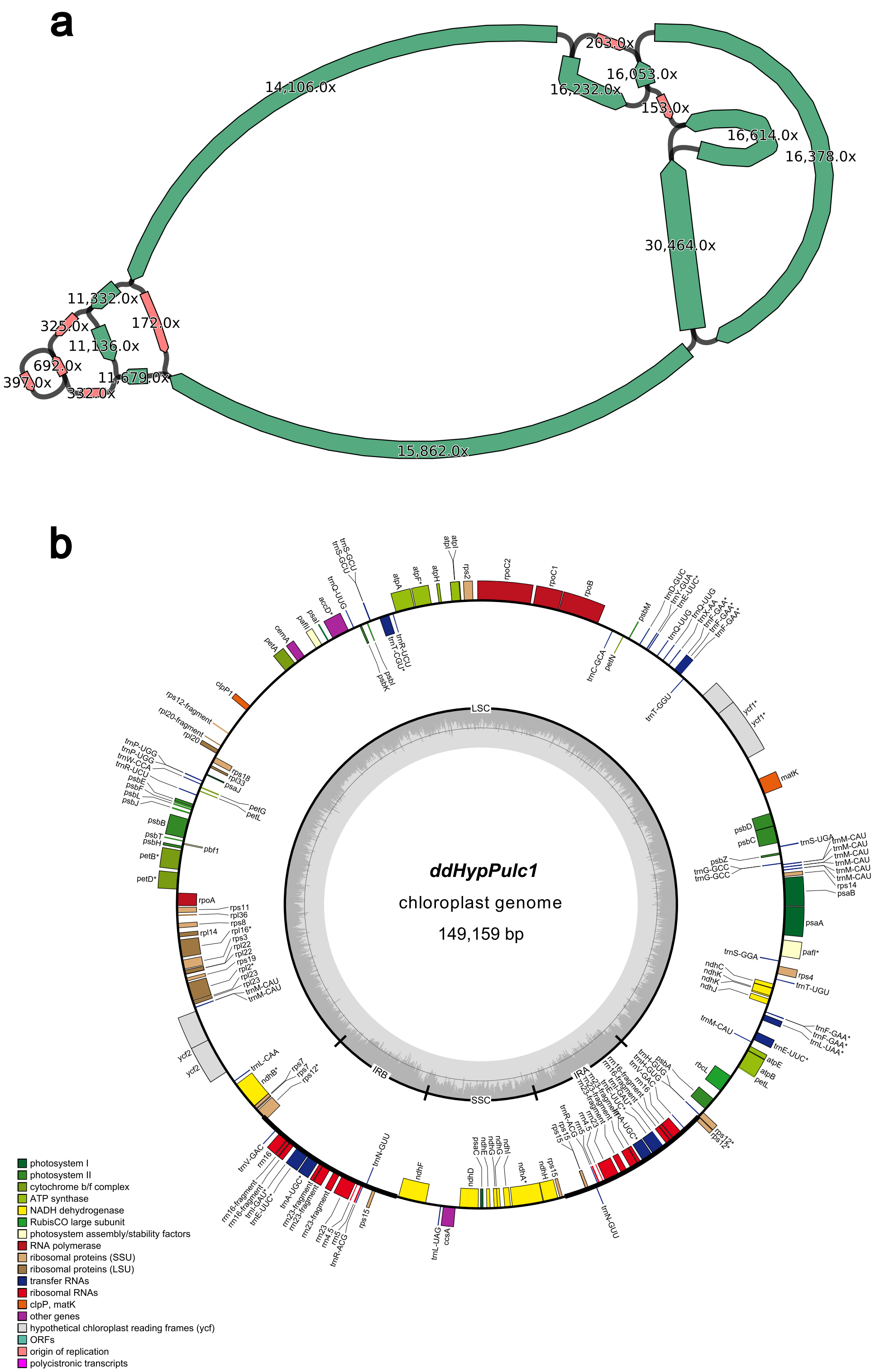

Fig S7. Plastome structure of *Hypericum pulchrum*. a, the genome assembly graph. The numbers on the bar indicate the sequence length and coverage. Sequences in green represent a path in the graph for the most abundant haplotype, while sequences in red represent heteroplasmy. **b**, the genome annotation. The assembly graph was produced using Bandage with additional manual adjustments. The annotation was performed using GeSeq and visualised with OGDRAW.

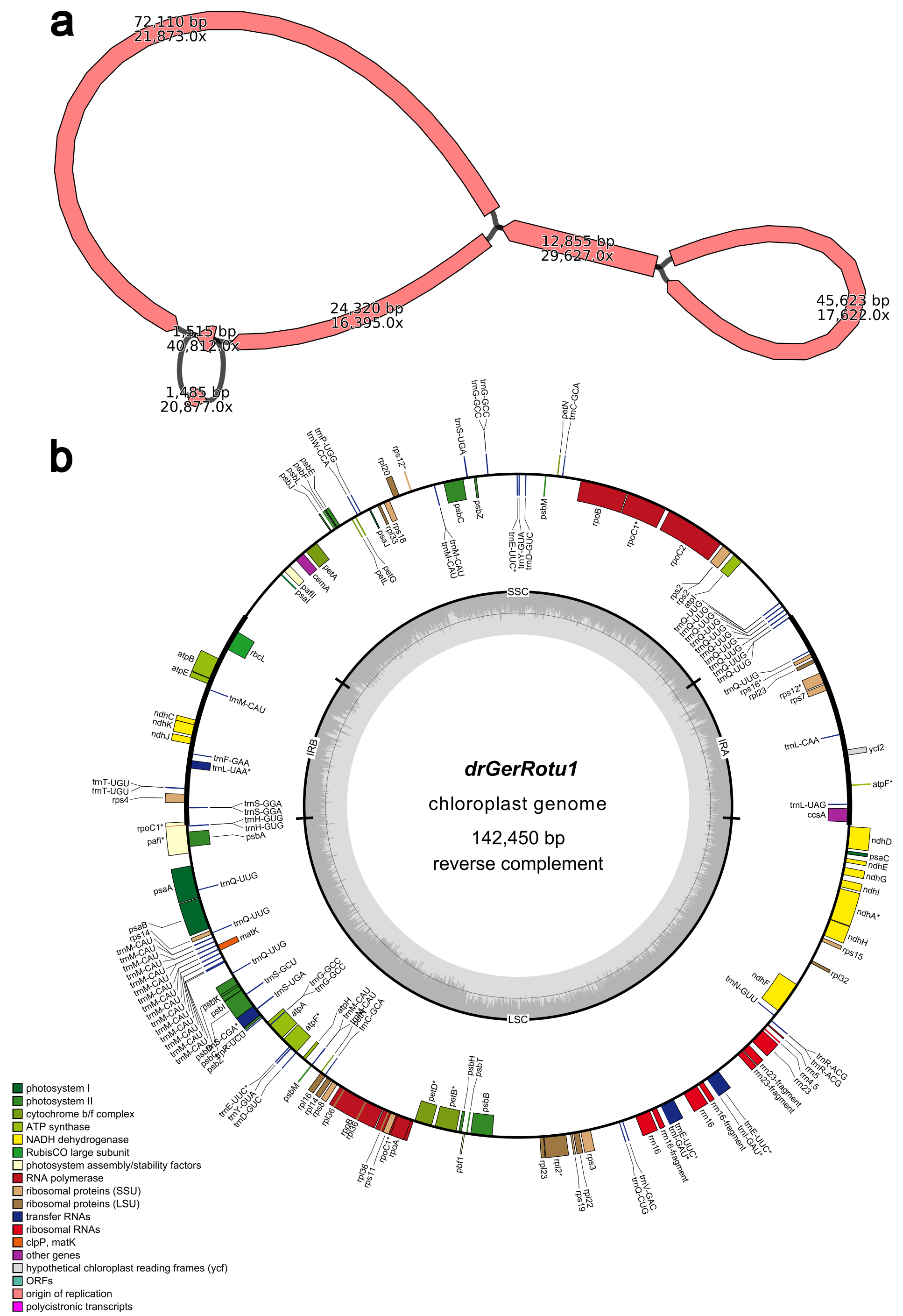

Fig S8. Plastome structure of *Geranium rotundifolium*. a, the genome assembly graph. The numbers on the bar indicate the sequence length and coverage. **b**, the genome annotation. The assembly graph was produced using Bandage with additional manual adjustments. The annotation was performed using GeSeq and visualised with OGDRAW.

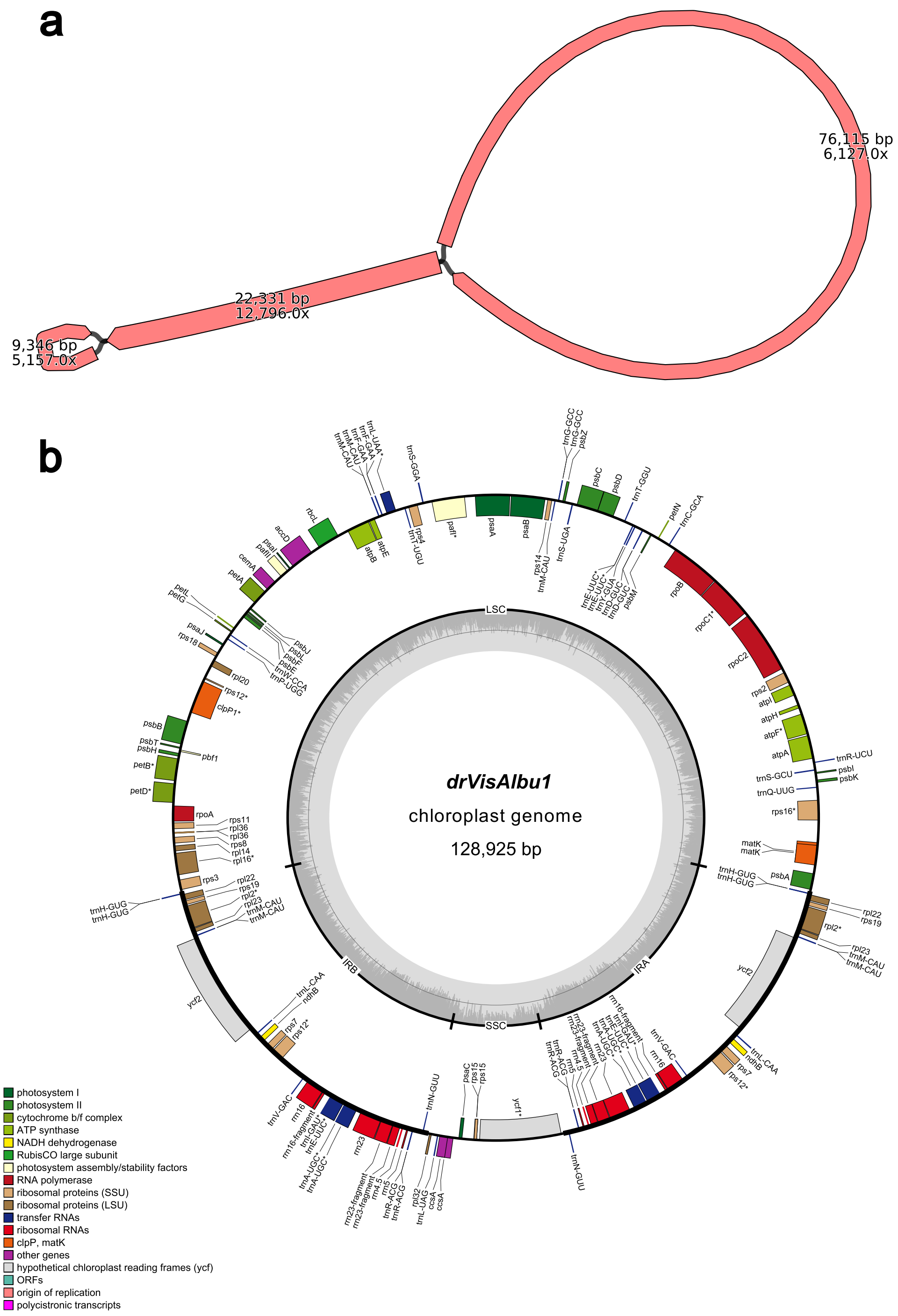

Fig S9. **Plastome structure of *Viscum album*.** **a**, the genome assembly graph. The numbers on the bar indicate the sequence length and coverage. **b**, the genome annotation. The assembly graph was produced using Bandage with additional manual adjustments. The annotation was performed using GeSeq and visualised with OGDRAW.

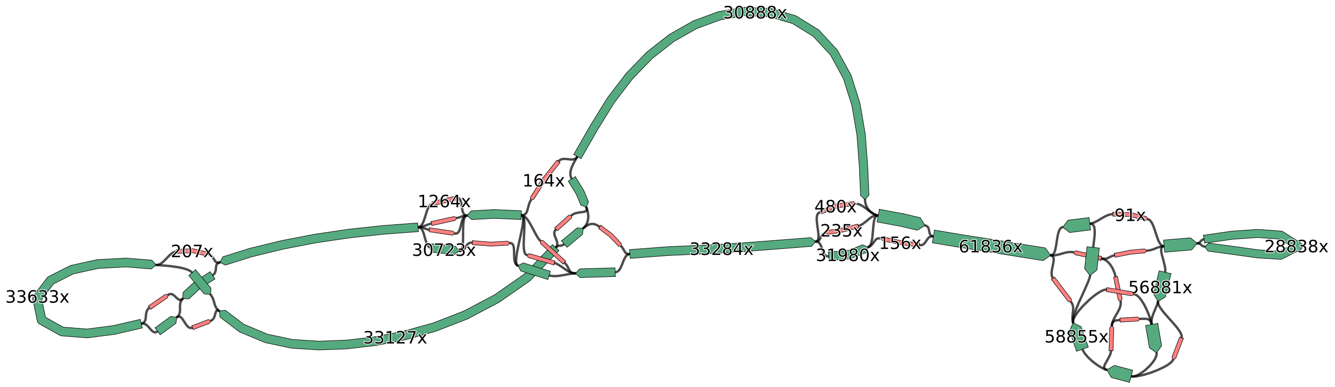

Fig S10. Plastome structure of *Hypericum perforatum*. In the graph, the width of each bar is proportional to the sequence coverage. The number on the bar indicates the actual coverage of the sequence, with some omitted for clarity. Sequences in green represent a path in the graph for the most abundant haplotype, while sequences in red represent heteroplasmy. The assembly graph was produced using Bandage with additional manual adjustments.

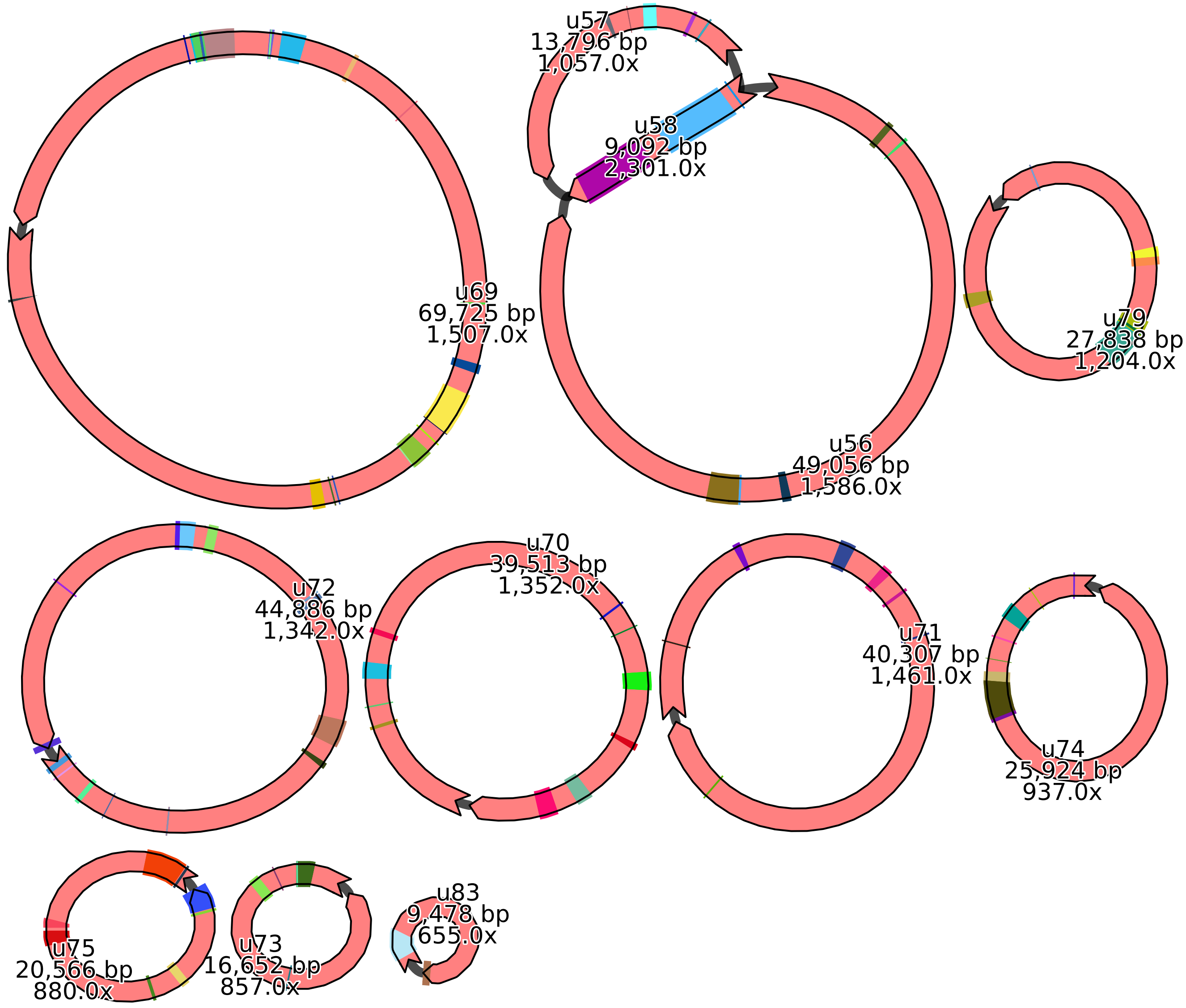

Fig S11. Mitogenome assembly of *Galeopsis tetrahit*. The genome assembly consists of ten components, with coloured blocks over the bars representing annotated genes. The numbers on the bar indicate the sequence name, length and coverage. The assembly graph was produced using Bandage with additional manual adjustments.

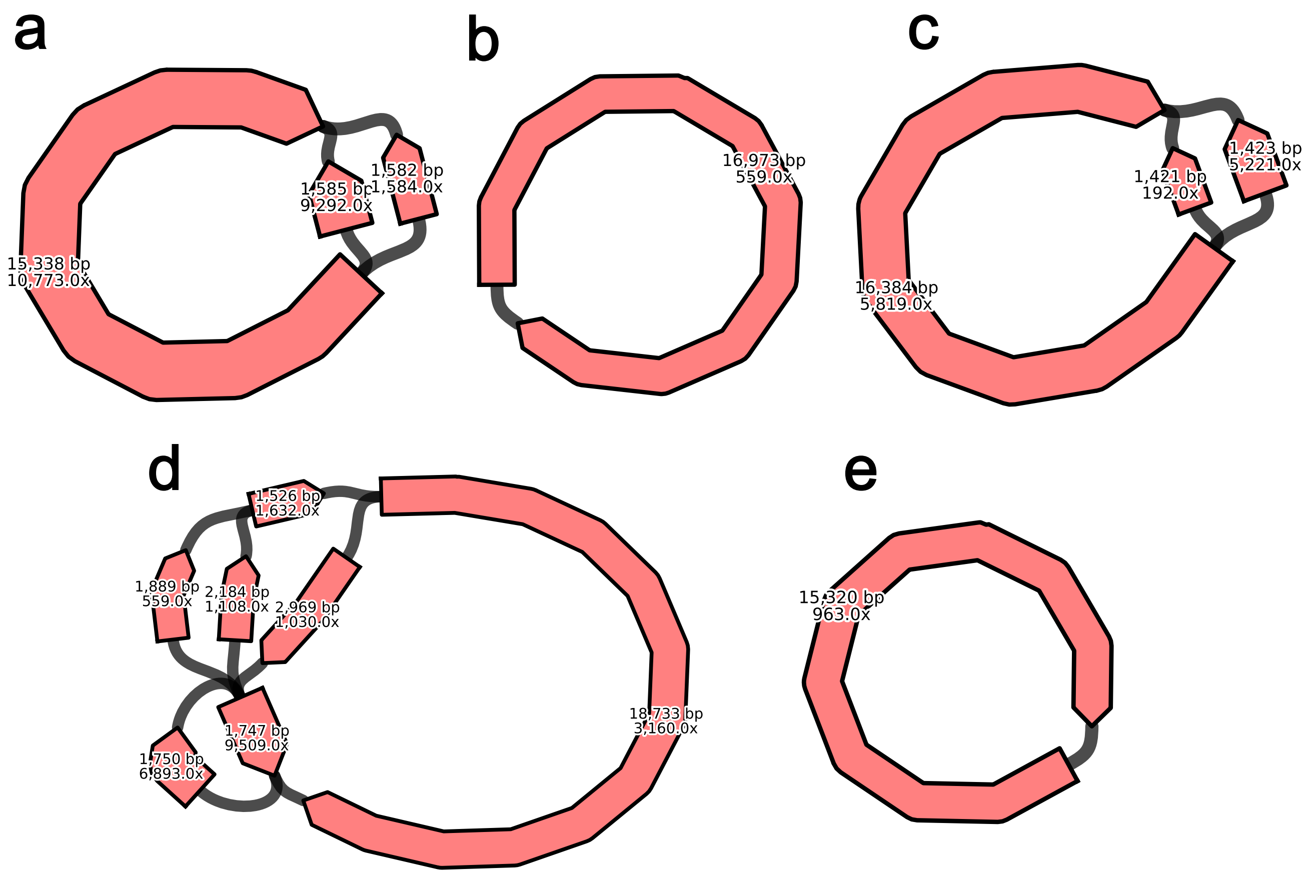

Fig S12. Mitogenome assembly of five animal species. a, *Micromys minutus* (TOLID: mMicMin1; ACCESSION: PRJEB72093). **b**, *Abramis brama* (TOLID: fAbrBra2; ACCESSION: PRJEB73975). **c**, *Anas platyrhynchos* (TOLID: bAnaPla2; ACCESSION: PRJEB76742). **d**, *Podarcis gaigeae* (TOLID: rPodGai1; ACCESSION: PRJEB76108). **e**, *Erebia medusa* (TOLID: ilEreMedu1; ACCESSION: PRJEB76717). The numbers on the bar indicate the sequence length and coverage. The assembly graph was produced using Bandage with additional manual adjustments.
